# Teaching Beliefs on Developmentally Appropriate Practice Among Chinese Preschool Teachers: The Role of Personality

**DOI:** 10.3389/fpsyg.2019.02822

**Published:** 2019-12-13

**Authors:** Paul Yau-Ho Wong

**Affiliations:** School of Arts and Humanities, Tung Wah College, Mongkok, Hong Kong

**Keywords:** Developmentally Appropriate Practice, personality, preschool teachers, teaching beliefs, teaching practices

## Abstract

Research has shown that teachers’ personality is related to their teaching practices. However, few such research has been conducted in the preschool settings. This research adopted a cross-sectional quantitative approach to examine how preschool teachers’ personality related to their beliefs of Developmentally Appropriate Practice (DAP). 544 preschool teachers were randomly selected to complete Teacher Beliefs and Practices Survey and the Myer–Briggs Type Indicator (MBTI). Findings showed that the personality type profiles of preschool teachers are characterized predominantly by Sensing-Feeling-Judging. Among all personality types, extraverted and intuitive teachers tended to show higher scores in DAP beliefs and practice than that of the introverted teachers. Analyses from regression findings also indicated that the Extroversion-Introversion dimension predicted significantly teachers’ beliefs and practices of DAP. In addition, teachers’ teaching experiences in association with personality predicted their DAP practices, suggesting that there was an combined effect of teachers’ personality and work experience in adopting DAP. Implication for school managers to provide professional development to support teachers in understanding and practicing DAP in preschools are discussed.

## Introduction

Quality education is conducive to young children’s later development (Early et al., 2007) and learning motivation in later schooling (Magnuson et al., 2007). According to the National Association for the Education of Young Children [NAEYC] (2013), quality early childhood education builds on the Developmentally Appropriate Practice (DAP) that “involves teachers meeting young children where they are (by stage of development), both as individuals and as part of a group; and helping each child meet challenging and achievable learning goals.” Evidences have shown that the DAP approach has positive impact on young children’s learning and development. As such, children found their learning more meaningful in schools that implemented the DAP approach (Lee and Lin, 2013) and displayed better psychological well-being outcomes in terms of self-competency (Jambunathan, 2012) and self-efficacy and social skills (Marcon, 2002). In turn, teachers tend to be more job satisfied upon observations of students’ authentic learning (Shoshani and Eldor, 2016). Taken together, the DAP approach supports the fact that it has positive impact on both young children and teachers.

## Theoretical Framework

According to Bronfenbrenner’s (1979) ecological theory, teachers’ beliefs and attitudes are affected by multi-factors, including external factors such as ethnic cultural beliefs and education systems and internal factors such as personality and personal beliefs of an individual. Moreover, dynamic inter-connections exist among these systems. Before the handover of sovereignty to China, Hong Kong was a British colony characterized by a culture of “East meets West.” After the handover, this cultural diversity has remained despite the fact that 92% of the population are ethnic Chinese (Census and Statistics Department, 2016). Although the Hong Kong government has intended to cultivate child-centeredness in preschool education since the past two decades, preschools tend to adopt traditional Chinese cultural values such as demands for student compliance and memorization which are regarded as barrier against child-centeredness (Rao et al., 2016). Comparable to child-centered pedagogy such as the Project approach and High Scope approach, the DAP addresses children’s “social and cultural contexts” (Copple and Bredekamp, 2009) that is argued to allow more contextual flexibility for teachers to meet children’s learning needs and thus believed to mismatch, at least to some extent, with the local cultural values.

Setting aside the influence of cultural values, internal personality has been found to be specifically attuned to teaching beliefs and practices among pre-service teachers. Pre-service teachers are referred to those student teachers who undergo full-time training at a teacher education institution in order to attain the Qualified Teacher Status from the Education Bureau (2015), whereas in-service teacher are those qualified teachers who works currently in preschools. Ripski et al. (2011) found that the Extroversion trait of pre-service teachers negatively predicted student–teacher interaction quality, whereas Agreeableness and Openness were found to be related to teachers’ beliefs about students’ diversity (Garmon, 2004; Unruh and McCord, 2010). Similarly, Decker and Rimm-Kaufman (2008) indicated that open and liberal teachers tended to encourage more students’ creativity and diverse thinking in classrooms. In preschool settings, McMullen (1999) found that preschool teachers characterized by an internal locus of control tended to prefer the DAP approach. Unfortunately, it is imperative to note that the majority of research that has been studied was mainly conducted among elementary and secondary teachers in the West; few such research indeed has been conducted in preschool settings, especially in the Chinese context.

This research adopts the Myers–Briggs Type Indicator (MBTI, Myers and McCaulley, 1985) to assess teachers’ personality types. While most personality inventories such as the five-factor model of personality (FFM, McCrae and Costa, 1999) lack a theoretical framework, the MBTI adopts Jung’s theory of psychological types (Jung, 1921/1971) as its theoretical background. The MBTI measures four pairs of personality dimensions, including the Extroversion-Introversion, Sensing-Intuition, Feeling-Thinking, and Judging-Perceiving dimensions (see Wong, 2012 for details). Jung (1921/1971) advocated that personality types are analogous to a person’s preferences for left-handedness or right-handedness. In other words, within the same personality dimension, one personality type can be dominant over the other personality types. Although the MBTI has been criticized of its operationalization of dichotomized type, it is a popular personality instrument (Furnham et al., 2003). Among all MBTI personality type dimensions, the Extroversion-Introversion, Sensing-Intuition and Feeling-Thinking dimensions are believed to be related to openness and sensitivity to children’s learning and development needs. Research has shown that Extroversion, openness, agreeableness and conscientiousness of the FFM were positively related to extroversion, intuition, feeling and judging personality types of the MBTI, respectively (McCrae and Costa, 1989; Furnham et al., 2003). In terms of vocational personality, Wong and Zhang (2013) found that the personality types of preschool teachers are predominantly characterized by the Extroversion (60%), sensing (91%), feeling (65%), and Judging (79%) types. While Wong’s study mainly examined in-service teachers, this research aimed to examine both pre-service and in-service teachers’ personality types. Apart from personality, other teachers’ personal characteristics such as age and field experiences (Kim, 2011), learning experiences from past schooling (Saban, 2003), school type and policy (Friedrich and Hron, 2011), and teaching specialty (Llurda and Lasagabaster, 2010) have been shown to impact on teachers’ teaching beliefs. These variables are anticipated to contribute to teachers’ teaching beliefs.

Three research questions and corresponding hypotheses are proposed:

(1) Are there differences in personality types between in-service and pre-service preschool teachers?
(2) Which personality types will show higher preferences of DAP beliefs and practices?
  Hypothesis 1: Teachers of the extraverted types would show higher scores of DAP than teachers of the introverted types, respectively.
  Hypothesis 2: Teachers of the intuitive types would show higher scores of DAP than teachers of the sensing types.
(3) Do teachers’ demographic characteristics contribute to beliefs and practice of DAP?

## Method

### Design

This research adopted a cross-sectional quantitative approach.

### Sample

A random sample of 1180 questionnaires package were distributed and eventually 544 returned (response rate: 47.2%). Though the response rate was moderately low, it was comparable to that of the average figure reported by Baruch (1999). Of all 544 participants, 530 (97.4%) and 14 (2.6%) were female and male, 156 and 401 were Bachelor of Education (B. Ed, Early childhood education) degree students (in-service) and Higher Diploma (HD, Early childhood education) (pre-service), respectively. All participants had prior knowledge about DAP from their learning curriculum at The Education University of Hong Kong. Their mean age and mean total teaching experience were 24.18 years (age range from 18 to 53, median age = 23) and 2.77 years, respectively.

### Measures

#### The Teacher Beliefs and Practices Survey (Burts et al., 2000; Leung, 2012)

The Teacher Beliefs and Practices Survey (3–5 years old), originally designed by Burts et al. (2000) and then modified by Leung (2012), was adopted to measure preschool teachers’ beliefs and practices. The modified versions were back translated to Chinese and then reviewed after a pilot trial by the researcher.

##### The teacher beliefs survey (TBS, Leung, 2012)

The TBS is a 19-item scale that measures teachers’ beliefs about DAP, with satisfactory reliability and validity. Leung demonstrated that the TBS comprised three factors, namely the Developmentally Appropriate Belief (DAPB, 9 items), the Developmentally inappropriate Practice Belief (DIPB, 6 reverse items), and the Family, Culture and Inclusion (FCI, 4 items) that matched with the original versions. After reverse scoring of the DIPB, participants that have higher scores show stronger beliefs in DAP. Kim (2005) reported that the Cronbach’s alphas of DAPB, DIPB, and FCI were 0.85, 0.82, and 0.81, respectively, whereas in the present research were 0.67, 0.66, and 0.69. All items are written in a 5-point Likert format: 1 = Not at all important, 2 = Not very important, 3 = Fairly important, 4 = Very important, and 5 = Extremely important. Sample items for each subscale are:

*For DAPB*: It is ( ) to read stories daily to children, individually and/or on a group basis.
*For DIPB: It is* ( ) *for teachers to regularly use punishments and/or reprimands when children aren’t participating.*
*For FCI: It is* ( ) *for parents/guardians to be involved in ways hat are comfortable for them.*

##### The instructional activities scale (IAS, Leung, 2012)

The IAS is a 19-item scale that assesses teachers’ practices about DAP, with satisfactory reliability and validity. Leung demonstrated that the IAS comprised four factors namely, the Developmentally Appropriate Practices Principles (DAPP, 8 items), Developmentally Appropriate Practices Activities (DAPA, 5 items), Developmentally Inappropriate Practices Activities (DIPA, 4 reverse items), and Developmentally Inappropriate Classroom Practices (DICP, 2 reverse items) that matched with the original versions. After reverse scoring, participants that have higher scores show stronger beliefs in related appropriate practices. In addition, Kim (2005) reported that the Cronbach’s alphas of DAPP, DAPA, DIPA, and DICP were 0.82, 0.76, 0.73, and 0.59, whereas in the present research were 0.69, 0.64, 0.65, and 0.67. All items are written in a 5-point Likert format, 1 = Almost never (less than monthly), 2 = Rarely (monthly), 3 = Sometimes (weekly), 4 = regularly (2–4 times a week), and 5 = Often (daily). Sample items for each subscale are:

*For DAPP: Solve real math problems using real objects*…
*For DAPA: Draw, paint, work with clay*,…
*For DIPA: Experiment with writing*…
*For DICP: Participate in whole-class, teacher-directed instruction*.

#### The MBTI—Form G (Myers and McCaulley, 1985; Myers et al., 2003)

The MBTI is a self-report and forced-choice inventory that comprises four personality dimensions, including the Extroversion-Introversion (21 items), Sensing-Intuition (26 items), Thinking-Feeling (23 items), and Judging-Perceiving (24 items). For each item, the respondents are allowed to choose either one or two options that best reflected their feelings and attitudes in daily life. The scoring instruction of MBTI in this research adopts the continuous scoring method (Myers and McCaulley, 1985). For each personality dimension (e.g., Extroversion-Introversion), a lower score indicates the dominance of the first personality type (i.e., Extroversion) over the second personality type (i.e., Introversion). The MBTI has been widely used (Furnham et al., 2003) and shows acceptable reliability (Capraro and Capraro, 2002) and validity (Carlson, 1985). In Hong Kong, Wong and Zhang (2013) reported that the Cronbach’s alphas for the four personality dimensions of Extroversion-introversion, sensing-intuition, thinking-feeling, and judging-perceiving were 0.74, 0.66, 0.65, and 0.69, respectively, whereas in the present research were 0.76, 0.62, 0.72, and 0.67. Sample items for each dimension are:

*For extraversion-introversion: Are you usually (A) a* “*good mixer*,” *or (B) rather quiet and reserved?*
For sensing-intuition: Do you usually get along better with (A) imaginative people, or (B) realistic people?
For thinking-feeling: Are you more careful about (A) people’s feelings, or (B) their rights?
For judging-perceiving: When you go somewhere for the day, would you rather (A) plan what you will do and when, or (B) just go?

### Data Collection

Prior to data collection, ethical approval was obtained from the Research Ethical Committee at The Education University of Hong Kong. A random class list of students from B. Ed (Part-time) and HD (Full-time) was generated from the computer. For each class, concerned lecturers were approached by the research assistant and explained the purpose of research. A student list that showed those randomly selected students for that class was provided to the lecturer for dispatching the questionnaires package. Each package of questionnaires included the consent letter, the demographic sheet, the TBS, the IAS, the MBTI, and a returned envelope. All inventories would take 40 min to finish. Students were asked to complete the questionnaires at home. After completing the questionnaires, students were requested to post back to the researcher using the returned envelope. Reminder emails were sent to selected students 2 days and 4 days after receiving the questionnaires package.

### Data Analyses

For research question 1, the frequency distributions (in percent) was used to show and compare the dominance of personality types between teachers from the two Programmes. Thus, the higher the percentage, the larger the dominance. For research question 2, independent *t*-test was used to examine difference(s) in the scores of teaching beliefs and practices between personality types. For research question 3, the linear regression was used that included a two-step single-level analysis initially entering teachers’ demographic characteristics, followed by the addition of personality dimensions factors in Step 2. A negative beta value indicates the dominance of the first personality type over the second one and vice versa. The rationale was to examine the differential contributions made by teachers’ demographics such as year of working experience and age, and four dimensions of personality types on teachers’ DAP scores.

## Results

As shown in Table 1, findings indicated that the personality profiles of pre-service (HD) and in-service (B. Ed) teachers were similar in each personality dimension and characterized, respectively, by the dominance of Extroversion (52.6%)-Sensing (78.4%)-Feeling (62.0%)-Judging (82.6%) and Extroversion (51.4%)-Sensing (86.7%)-Feeling (64.0%)-Judging (83.4%), respectively.

**TABLE 1:** Distribution of kindergarten teachers’ personality types from B. Ed and HD programmes.

**Personality**	**B. Ed (*n* = 331)**	**HD (*n* = 213)**	**B. Ed + HD (*N* = 544)**
*Extroversion* (*E*)	170 (51.4)	112 (52.6)	282 (51.8)
Introversion (I)	161 (48.6)	101 (47.4)	262 (48.2)
*Sensing (S)*	287 (86.7)	167 (78.4)	454 (83.5)
Intuition (N)	44 (13.3)	46 (21.6)	90 (16.5)
Thinking (T)	119 (36.0)	81 (38.0)	200 (36.8)
*Feeling (F)*	212 (64.0)	132 (62.0)	344 (63.2)
*Judging (J)*	276 (83.4)	176 (82.6)	452 (83.1)
Perceiving (P)	55 (16.6)	37 (17.4)	92 (16.9)

*B. Ed, Bachelor of Education (Early childhood education) Part-time programme, HD (ECE), Higher Diploma (Early childhood education) Full-time programme. Personality types in italics are predominant.*

Because both in-service and pre-service teachers indicated very similar patterns of personality types, the data were combined for further analyses. As shown in Table 2, Extraverted teachers showed higher scores of TBS (*t* = 2.50, *p* < 0.01), DAPB (*t* = 2.64, *p* < 0.01), DAPP (*t* = 1.99, *p* < 0.05) and DAPA (*t* = 2.59, *p* < 0.01) than that of the Introverted teachers. Intuitive teachers displayed higher scores of TBS (*t* = −2.87, *p* < 0.01), DIPB (*t* = −2.16, *p* < 0.05), and DICP (*t* = −2.89, *p* < 0.01) than that of the Sensing teachers. Teachers of the Perceiving types showed higher scores of DAPB (*t* = −1.99, *p* < 0.05) than teachers of the Judging types.

**TABLE 2:** Personality types and it associated mean scores on Developmentally Appropriate Practices.

	**E**	**I**	***t*-value**	**S**	**N**	***t*-value**	**T**	**F**	**J**	**P**	***t*-value**
**TBS**											
DAPB	35.1	34.4	2.64^∗∗^	34.6	35.3		34.7	34.8	34.6	35.4	−1.99^∗^
DIPB	11.5	11.4		11.4	12.0	−2.16^∗^	11.4	11.5	11.5	11.5	
FCI	16.0	15.7		15.7	16.2		15.7	15.9	15.8	16.1	
TBS total	62.7	61.5	2.50^∗∗^	61.9	63.6	−2.87^∗∗^	61.9	62.3	61.9	63.1	
**IAS**											
DAPP	30.7	30.0	1.99^∗^	30.4	30.2		30.7	30.2	30.4	30.3	
DAPA	18.8	18.3	2.59^∗∗^	18.5	18.8		18.5	18.6	18.5	18.7	
DIPA	7.2	7.5		7.3	7.5		7.2	7.4	7.3	7.4	
DICP	3.4	3.4		3.3	3.8	−2.89^∗∗^	3.3	3.4	3.4	3.2	
IAS Total	60.2	59.4		59.7	60.5		59.8	59.8	59.8	59.8	

*TBS, teacher beliefs survey, DAPB, Developmentally Appropriate Belief; DIPB, Developmentally inappropriate Belief; FCI, Family, Culture and Inclusion; IAS, Instructional Activities Scale; DAPP, Developmentally Appropriate Practices Principles; DAPA, Developmentally Appropriate Practices Activities; DIPA, Developmentally Inappropriate Practices Activities; DICP, Developmentally Inappropriate Classroom Practices, E, Extroversion; I, introversion; S, Sensing; N, intuition; T, thinking; F, feeling; J, judging; P, perceiving. ^∗∗^*p* < 0.01; ^∗^*p* < 0.05.*

The regression findings as shown in Table 3, indicated that the Extroversion-Introversion dimension (model 2: β = −0.11, *t* = −2.46, *p* < 0.05) predicted DAPB as a single predictor, whereas the Sensing-Intuition dimension (model 2: β = 0.20, *t* = 1.94, *p* = 0.05) was significant predictor of TBS total. For the IAS, teaching experience predicted significantly DAPP (model 1: β = 0.29, *t* = 4.29, *p* < 0.001), DAPA (model 1: β = 0.16, *t* = 2.21, *p* < 0.05), DIPA (model 1: β = −0.17, *t* = −2.44, *p* < 0.05) and IAS total (model 1: β = 0.17, *t* = 2.39, *p* < 0.05). The Extroversion-Introversion was a significant predictor of DAPP (model 2: β = −0.13, *t* = −3.13, *p* < 0.001), DAPA (model 2: β = −0.14, *t* = −3.14, *p* < 0.001), DIPA (model 2: β = 0.09, *t* = 2.15, *p* < 0.01), and IAS total (model 2: β = −0.09, *t* = −2.03, *p* < 0.05) when teaching experience remained significant. The Sensing-Intuition dimension (model 2: β = 0.16, *t* = 3.42, *p* < 0.001), and the Judging-Perceiving dimension (model 2: β = −0.10, *t* = −1.98, *p* < 0.05) predicted DICP, respectively.

**TABLE 3:** Linear regression of predictors of teaching beliefs and practices.

	**DAPB**	**DIPB**	**FCI**	**TBS total**	**DAPP**	**DAPA**	**DIPA**	**DICP**	**IAS total**
*R*^2^_Total_	0.02	0.01	0.01	0.03	0.13	0.04	0.03	0.03	0.05
*R*^2^_Model 1_	0.00	0.00	0.00	0.00	0.05^∗∗∗^	0.01	0.01^∗^	0.00	0.02
β_age_	–0.03	0.08	0.04	0.03	–0.08	–0.11	0.11	–0.05	–0.05
β_t_exp_	0.05	–0.04	0.01	0.02	0.29^∗∗∗^	0.16^∗^	−0.17^∗^	0.04	0.17^∗^
*R*^2^_Model 2_	0.02^∗^	0.01	0.01	0.03	0.08^∗∗∗^	0.03^∗∗∗^	0.02	0.03^∗^	0.03
β_age_	–0.02	0.08	0.04	0.04	–0.07	–0.08	0.09	–0.07	–0.05
β_t_exp_	0.04	–0.03	0.01	0.02	0.27^∗∗∗^	0.14^∗^	−0.15^∗^	0.07	0.16^∗^
β_E–I_	−0.11^∗^	0.02	–0.05	–0.08	–0.13^∗∗∗^	–0.14^∗∗∗^	0.09^∗∗^	0.01	−0.09^∗^
β_S–N_	0.05	0.08	0.07	0.10^∗^	0.03	0.03	0.03	0.16^∗∗∗^	0.08
β_T–F_	–0.01	0.03	0.02	0.02	–0.07	–0.01	0.03	0.06	–0.02
β_J–P_	0.03	0.01	–0.02	0.02	–0.07	–0.02	–0.01	−0.10^∗^	–0.06

*TBS, teacher beliefs survey, DAPB, Developmentally Appropriate Belief; DIPB, Developmentally inappropriate Belief; FCI, Family, Culture and Inclusion; IAS, Instructional Activities Scale; DAPP, Developmentally Appropriate Practices Principles; DAPA, Developmentally Appropriate Practices Activities; DIPA, Developmentally Inappropriate Practices Activities; DICP, Developmentally Inappropriate Classroom Practices; E-I, Extroversion-Introversion; S-N, Sensing-Intuition; T-F, Thinking-Feeling; J-P, Judging-Perceiving; t_exp, teaching experience. ^∗∗∗^*p* < 0.001, ^∗∗^*p* < 0.01, ^∗^*p* < 0.05.*

## Discussion

This research aimed to examine how preschool teachers’ personality related to their beliefs of DAP. Findings showed that the personality type profiles of preschool teachers were characterized predominantly by Sensing-Feeling-Judging. Among all personality types, extroverted and intuitive teachers tended to show higher scores in DAP beliefs and practice. In addition, teaching experiences associated with personality predicted DAP practices. The followings discuss current findings and its implications to the field:

### Robustness of Preschool Teachers’ Personality Types

The frequencies of teachers’ personality types from the two different teacher training programmes are similar to a large extent, showing that preschool teachers are characterized predominantly by the personality types of Sensing-Feeling-Judging. The findings are comparable to that of the previous study by Wong and Zhang (2013). Taken together, consistent evidences have provided strong support to the robustness of preschool teachers’ personality types. According to Myers and McCaulley (1985), the personality types of Sensing-Feeling-Judging are characterized by preferences of following instructions in a structured work environment and caring for others but are not comfortable with applying DAP that taps on liberal and flexible thinking. Thus, this robustness of personality types that indicates the relative weakness of openness and flexibility among preschool teachers is argued to act as a barrier to implement the DAP in schools. Wong and Zhang (2013) argued that intuitive teachers show leadership capability and incline to be more comfortable with planning and implementing creative curriculum in preschool settings. Furthermore, it is interesting to note that the frequency of intuitive pre-service teachers (21.6%) was higher than that of in-service teachers (13.3%), suggesting that there is greater propensity of the former to adopt the DAP beliefs, whereas more professional training programmes in DAP are necessary for in-service teachers. As such, teacher training institutions are recommended to strengthen pre-service teachers’ understanding of DAP and enhance self-reflection with respect to their personality characteristics.

### Extraverted and Intuitive Teachers Tend to Show Stronger Beliefs in DAP

As expected, extraverted teachers and intuitive teachers tended to exhibit stronger beliefs in DAP than that of the introverted teachers and sensing teachers, respectively (a lower score indicates the dominance of the first personality type (i.e., Extroversion) over the second personality type (i.e., Introversion) and vice versa). Therefore, both hypotheses are accepted. These findings support that notion that teachers’ personality that are characterized by openness and flexibility are more willing to adopt and practice the DAP. In preschools, young children tend to find teachers who are communicative and flexible more approachable (Saracho, 2003). It follows that extraverted and intuitive teachers are more inclined and able than that of their introverted and sensing counterparts to implement DAP in classrooms. As such, it may be desirable for school principals to identify teachers of extraverted and intuitive personality that match with the characteristics of the DAP for recruitment. In addition, school managers are recommended to provide individualized professional development programs that focus on understanding and practicing DAP to all teachers in their schools.

### Combined Effect of Teaching Experience and Personality on DAP Beliefs

Current findings also indicated that there was a joint effect of personality (i.e., extraverted and intuitive personality types) and teaching experiences that contributed to teachers’ beliefs and practice in DAP. It is imperative to note that though the majority of preschool teachers are of the sensing types that tend not to prefer the beliefs and practices of DAP, their teaching experiences are believed to minimize this limitation to a certain extent. In this sense, positive experiences about DAP are believed to be able to enhance teachers’ belief and subsequent practices (Murphy et al., 2015; Smith, 2015). The implication is that school principals and teacher training institutions can provide ongoing professional development training programs for teachers to construct their own successful teaching experiences of DAP such that teachers are able to develop substantial amount of positive experiences to cultivate and maintain their DAP beliefs. In addition, school managers are suggested to maintain the stability of teaching team so that experienced and intuitive teachers (Rushton et al., 2007) are able to lead the development of DAP in schools.

## Limitations, Recommendations, and Conclusion

This research adopted a cross-sectional design such that there was no causal relationship among variables to be generated. Future research is recommended to use qualitative approach such as semi-structured interviewing. Though random sampling was adopted, the sample was based on only one tertiary institution and female dominated such that any generalization of findings should be cautious. Future studies are recommended to adopt a longitudinal research design that has the strength of making inferences from cause-effect relationships among research variables. In addition, multiple measures of teachers’ responses by employing physiological indicators such as cortisol, heart rate, and body temperature, as objective measurements of participants’ levels of subjective well-being should be considered.

In conclusion, preschool teachers are characterized by a robustness of Sensing-Feeling-Judging personality types. In particular, extraverted and intuitive teachers tend to show stronger beliefs in DAP. Nonetheless, teaching experience is a compensatory factor in enhancing the DAP beliefs. School principals and teacher education institutions are recommended to provide appropriate professional development programmes to promote the readiness of preschool teachers in adopting the DAP approach in preschools.

## Data Availability Statement

All datasets generated for this study are included in the article/supplementary material.

## Ethics Statement

The studies involving human participants were reviewed and approved by The Education University of Hong Kong. The patients/participants provided their written informed consent to participate in this study.

## Author Contributions

The author confirms being the sole contributor of this work and has approved it for publication.

## Conflict of Interest

The author declares that the research was conducted in the absence of any commercial or financial relationships that could be construed as a potential conflict of interest.
